# Modified Silica Particles Coated with Cu-Al Layered Double Hydroxide for Phosphate and Arsenate Removal in Water Treatment

**DOI:** 10.3390/molecules30102138

**Published:** 2025-05-13

**Authors:** Andrija Savić, Marija M. Vuksanović, Marjetka Savić, Nataša Knežević, Aleksandra Šaponjić, Svetlana Ilić, Adela Egelja

**Affiliations:** 1 Department of Chemical Dynamics and Permanent Education, “VINČA” Institute of Nuclear Sciences, National Institute of the Republic of Serbia, University of Belgrade, Mike Petrovića Alasa 12-14, 11351 Belgrade, Serbia; marija.vuksanovic@vin.bg.ac.rs (M.M.V.); metk@vin.bg.ac.rs (M.S.); natasa.knezevic@vin.bg.ac.rs (N.K.); adela@vin.bg.ac.rs (A.E.); 2 Department of Materials, “VINČA” Institute of Nuclear Sciences, National Institute of the Republic of Serbia, University of Belgrade, Mike Petrovića Alasa 12-14, 11351 Belgrade, Serbia; acavuc@vin.bg.ac.rs (A.Š.); svetlanailic@vin.bg.ac.rs (S.I.)

**Keywords:** layered double hydroxides composites, adsorption process, GLYMO, phosphate, arsenate ions

## Abstract

Environmental pollution remains one of the most pressing challenges facing modern society, with the removal of toxic substances from water sources being of particular concern. In this study, a composite material was synthesized by combining Cu-Al layered double hydroxides (CuAl-LDHs) with modified silica particles, aiming to develop an efficient and environmentally friendly adsorbent for the removal of phosphate and arsenate ions from water. CuAl-LDH, with a Cu^2+^/Al^3+^ molar ratio of 2:1, was synthesized using the co-precipitation method in the presence of modified silica maintaining an LDH/SiO_2_ mass ratio of 2:1. The silica particles were functionalized with 3-glycidyloxypropyltrimethoxysilane (GLYMO) followed by modification with polyethyleneimine (PEI) to enhance their adsorption properties. X-ray diffraction (XRD) confirmed the successful deposition of CuAl-LDH on the silica surface, while scanning electron microscopy (SEM) revealed the porous structure of the silica and the uniform deposition of LDH. Adsorption experiments were performed to evaluate the removal efficiency of phosphate and arsenate ions under varying conditions. Equilibrium adsorption capacities, based on the Langmuir isotherm model, were determined to be 44.6 mg·g^−1^ for phosphate (PO_4_^3−^) and 32.3 mg·g^−1^ for arsenate (As(V)) at 25 °C. The sorption behavior was better described by the Freundlich isotherm model, which yielded K_F_ values of 15.4 L·mg^−1^ for phosphate and 13.9 L·mg^−1^ for arsenate. Both batch and kinetic experiments confirmed the high adsorption efficiency of the composite, demonstrating its potential as a promising material for water treatment applications.

## 1. Introduction

Phosphorus is an essential element for all living cells, playing a critical role in energy transport through adenosine triphosphate (ATP) and serving as an integral part of the structural frameworks of DNA and RNA. The element is widely used in various industries, most notably in the production of fertilizers. However, the release of phosphorus along with other agricultural pollutants into water or soil can cause serious environmental problems [1,2,3]. One of the most significant environmental effects of phosphorus pollution is eutrophication—a process where excess phosphorus leads to the overgrowth of aquatic plants and algae, depleting oxygen in water bodies and threatening aquatic ecosystems [4,5,6].

Arsenic, on the other hand, is a metalloid that occurs naturally in the Earth’s crust and is regarded as one of the most hazardous substances due to its high toxicity [7]. Prolonged exposure to arsenic, particularly through contaminated water, can result in serious health issues, including cancer, cardiovascular diseases, and neurological disorders [8]. In the environment, arsenic primarily exists in two oxidation states: arsenate (As(V)) and the more toxic arsenite (As(III)) [9]. Both forms are detrimental to living organisms, necessitating the development of efficient removal technologies to mitigate the risks posed by arsenic contamination. Yadav et al. [10] examined the adsorption of arsenate As(V) on MgFe(CO_3_)-LDH in the presence of different anions (CO_3_^2−^, Cl^−^, PO_4_^3−^, SO_4_^2−^, and NO_3_^−^). Their findings indicated that the presence of PO_4_^3−^ and CO_3_^2−^ ions significantly reduced the efficiency of As(V) removal [11].

A variety of techniques have been employed for the removal of phosphorus and arsenic from water, including coagulation, membrane filtration, flotation, flocculation, chemical precipitation, biological removal, crystallization, ion exchange, and adsorption [7,12,13,14]. Among these, adsorption has gained considerable attention due to its high efficiency, simplicity in preparation, minimal waste production, and relatively low cost [15,16]. Adsorption processes are widely utilized in water treatment for the removal of various pollutants due to their versatility, selectivity, and ease of operation. Furthermore, adsorption allows for faster regeneration of materials and offers greater adaptability compared with other techniques [4].

Layered double hydroxides (LDHs) are a versatile group of two-dimensional (2D) inorganic layered materials that have attracted significant interest due to their unique physical and chemical properties. These properties have enabled exceptional performance in a wide range of applications, including catalysis, photochemistry, electrochemistry, biotechnology, medicine, wastewater treatment via adsorption, and enzyme immobilization support; owing to their excellent enzyme retention capacity, LDHs help preserve enzyme activity and facilitate charge transport in immobilized systems. Common methods for synthesizing LDHs include co-precipitation, direct ion exchange, and rehydration techniques [17]. In recent decades, researchers have focused on developing new adsorbent materials that are not only efficient but also environmentally friendly, easy to synthesize, and economically viable. One such class of materials showing significant promise is LDHs. Often referred to as anionic clays, LDHs have attracted attention in various fields, including water treatment, biotechnology, polymer nanocomposites, and catalysis [18,19,20,21]. Structurally, LDHs can be described by the general formula [M^2+^_1−x_M^3+^_x_(OH)_2_]^x+^[A_x/n_·mH_2_O]^n−^, where M^2+^ and M^3+^ represent divalent and trivalent metal cations, respectively, x is the molar ratio of M^3+^/(M^3+^ + M^2+^), and A^n−^ denotes an anion that balances the positive charge within the hydrated interlayer regions [22]. The LDH structure consists of positively charged brucite-like layers, in which divalent metal cations are partially substituted by trivalent cations. This substitution creates a charge imbalance that is compensated by anions in the interlayer spaces [23,24]. This unique structure endows LDHs with high adsorption capacities for a wide range of pollutants, making them ideal candidates for water treatment applications. Ramazan Keyikoglu et al. presented the fundamental aspects and recent advances in phosphate removal using LDH-based materials containing Ca, Mg, and Zn in combination with trivalent cations such as Al and Fe. Notably, when LDH is supported on biochar—such as in MgAl-LDH@BC—the compo-site exhibits outstanding phosphate adsorption performance (up to approximately 410 mg·g^−1^) and significantly enhanced LDH dispersion [25]. Carbon-based materials provide a large specific surface area for LDH attachment, preventing aggregation and enhancing phosphate adsorption capacity compared with pure LDH. LDH–biochar (BC) composites have emerged as a novel and promising class of materials for the removal of various waterborne pollutants. Researchers such as Meili et al. [26] and Li et al. [27] have synthesized MgAl-LDH on biochar derived from bovine bone and sugarcane leaves, respectively, and successfully applied them to the adsorption of methylene blue and phosphate. Lartey-Young et al. [28] developed adsorbents by pyrolyzing bamboo biochar pre-impregnated with Cu–Zn–Fe LDH for atrazine removal. Similarly, Lesbani A. and his team investigated CuAl-LDH supported on rice husk biochar [29] and hydrochar derived from rambutan peel [30] for the adsorption of malachite green and methyl blue dyes [31].

The synthesis of polyethyleneimine (PEI)-based composite materials has garnered significant attention for the removal of toxic heavy metals, owing to their unique properties such as high metal-binding affinity, thermal stability, mechanical strength, and reusability potential. The presence of amine functional groups in PEI plays a crucial role in the efficient adsorption of both cationic and anionic heavy metal ions. To date, numerous studies have explored PEI-based composite materials for heavy metal removal [32]. In particular, PEI/Si nanomaterials have demonstrated high effectiveness in wastewater treatment, primarily due to the strong chelating properties of the amino groups in PEI [33].

In this study, we focus on the synthesis of a novel adsorbent material composed of CuAl-layered double hydroxide (CuAl-LDH) supported on modified porous silica particles. By integrating the adsorption capabilities of CuAl-LDH with the structural advantages of modified silica, the resulting composite material is designed to offer an efficient and environmentally sustainable solution for the removal of phosphate and arsenate from contaminated water sources. The CuAl-LDH@SiO_2_ composite was synthesized using the co-precipitation method, and its potential for phosphate and arsenate removal was systematically evaluated. This work contributes to the development of effective water purification technologies aimed at mitigating the harmful effects of these pollutants and supporting the protection of global water resources.

## 2. Results and Discussion

### 2.1. Characterization of CuAl-LDH@SiO_2_

**Figure 1:**
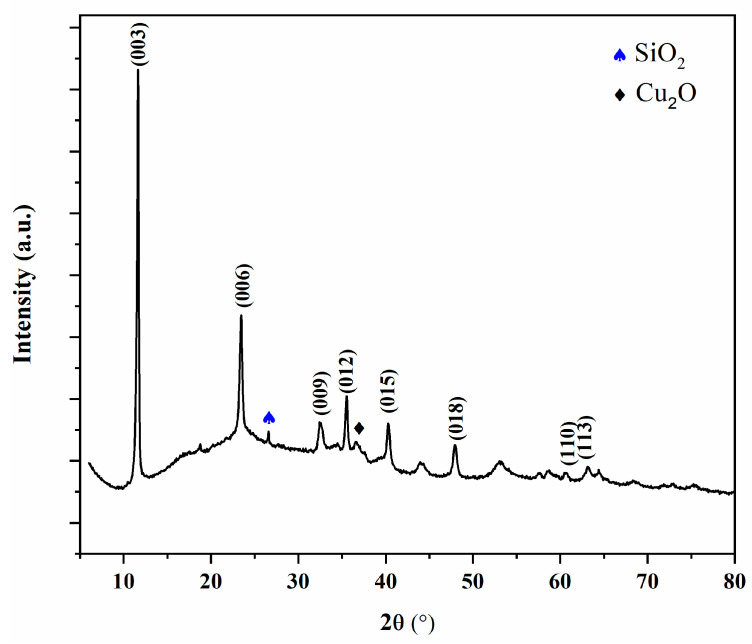
XRD of CuAl-LDH@SiO_2_ particles.

**Figure 2:**
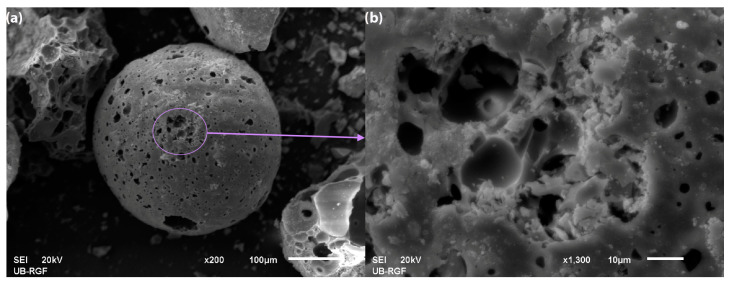
SEM images of (**a**) CuAl-LDH@SiO_2_ particles and (**b**) the framed part of the particle is enlarged.

The X-ray diffraction (XRD) pattern and surface morphology (SEM) of the synthesized material are presented in Figure 1 and Figure 2, respectively.

CuAl-LDH exhibited characteristic diffraction peaks at 11.7°, 23.4°, 35.5°, 47.8°, 58.7°, and 63.1°, corresponding to the (003), (006), (012), (015), (018), (110), and (113) crystal planes, respectively, indicating the layered structure of the material [34,35]. The reflections associated with the (003), (006), and (012) planes are sharp, intense, and symmetrical, suggesting high crystallinity of the sample. These strong low-angle peaks also indicate a significant interlayer spacing, a typical feature of well-ordered layered structures [4]. At higher 2θ values, broader and less intense peaks corresponding to the (015), (110), and (113) planes were observed, which is characteristic of the reduced long-range order in those directions. Additionally, the presence of a distinct diffraction peak corresponding to silica (SiO_2_) confirms the successful synthesis of the CuAl-LDH@SiO_2_ composite via the co-precipitation method. During synthesis, the addition of NaOH and a small amount of cuprous oxide (Cu_2_O) at approximately pH 6 led to the formation of Cu_2_O as a secondary phase [36].

After confirming the crystalline structure of the sorbent, scanning electron microscopy (SEM) analysis was performed to examine the morphological characteristics and surface features of the sample (Figure 2).

Figure 2a presents the structure of the synthesized sample at a larger scale. The image reveals the porous morphology of the silica particles, characterized by numerous cavities, indicating a high specific surface area. These pores enhance the surface activity of the material, thereby improving its ability to bind pollutants during the adsorption process. Figure 2b displays the same porous structure at a higher magnification, highlighting finer details of the material’s porosity. Clearly defined pores and their uneven distribution are visible, along with surface irregularities that confirm the successful deposition of CuAl-LDH on the silica support.

**Figure 3:**
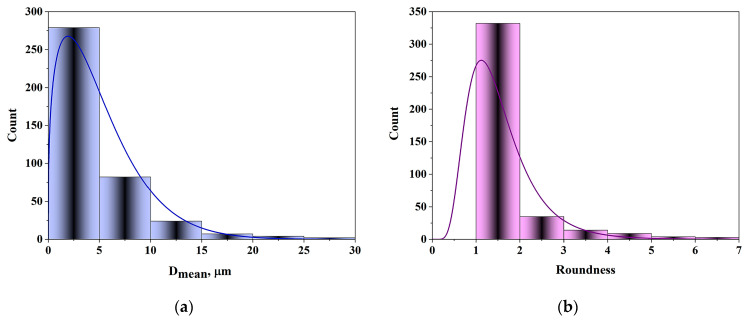
(**a**) Distribution of the diameter of pores; and (**b**) roundness of pores.

Pore diameters of the silica particles were measured using a series of SEM images. For pore size and sphericity estimation, image analysis was performed using Image-Pro Plus 6.0 software. The resulting distribution of pore diameters and sphericity is shown in Figure 3.

According to the pore size distribution, the majority of pores fall within the 2–5 µm range, while the sphericity values range from 1 to 2. These results indicate that most pores are approximately spherical in shape, with a portion displaying irregular geometries.

Energy-dispersive X-ray spectroscopy (EDS) analysis was performed over a broad surface area of the SEM sample to determine the elemental composition of the silica support on which CuAl-LDH particles were deposited. The EDS mapping confirmed the presence of elements characteristic of both the silica substrate and the CuAl-LDH phase. Specifically, silicon and oxygen represent the silica matrix, while copper and aluminum are attributed to the CuAl-LDH component. The elemental composition results are summarized in Table 1.

**Figure 4:**
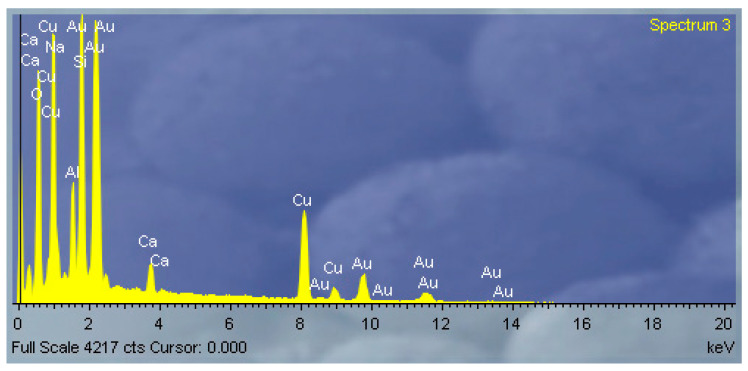
EDS mapping results of CuAl-LDH@SiO_2_ particles.

The CuAl-LDH synthesis was conducted to achieve a molar ratio of Cu:Al = 2:1, which was confirmed through EDS analysis based on atomic percentages. The Cu/Al/Si ratio indicates that the majority of the CuAl-LDH material is present on the surface of the silica particles. This confirms the successful deposition of CuAl-LDH onto the silica support. The elemental composition obtained from the EDS analysis (Figure 4) reveals the following atomic percentages: Si 11.27%, O 66.98%, Cu 12.14%, Ca 1.23%, and Al 5.21%.

### 2.2. Determination of Zero Point Charge and BET Analysis

**Figure 5:**
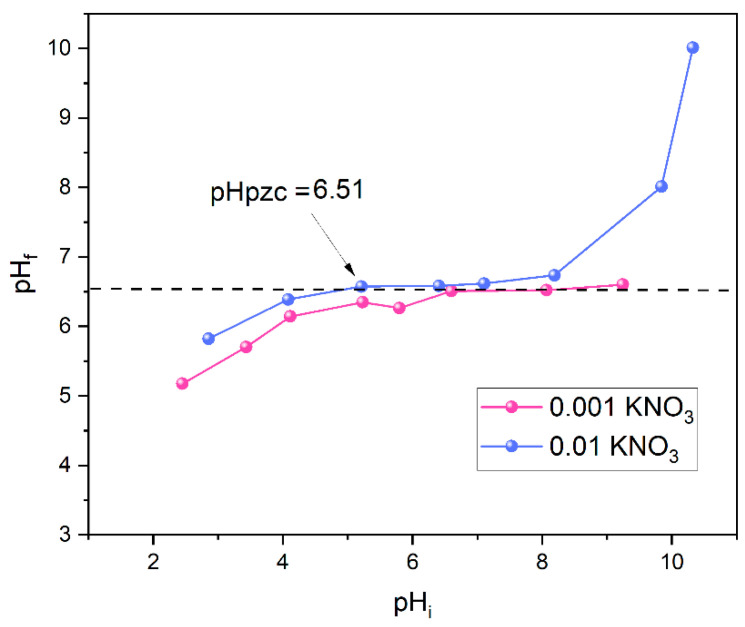
The final pH (pHf) versus initial pH (pHi) change for CuAl-LDH@SiO_2_.

The pH at the point of zero charge (pHpzc) for CuAl-LDH@SiO_2_ was found to be 6.51, as shown in Figure 5. The extent of the positive charge on the adsorbent surface at pH < pHpzc depends on the operating pH and surface characteristics, with minimal dependence on ionic strength. This result strongly suggests the high applicability of both adsorbents for the removal of anionic pollutants at pH < pHpzc [37].

**Figure 6:**
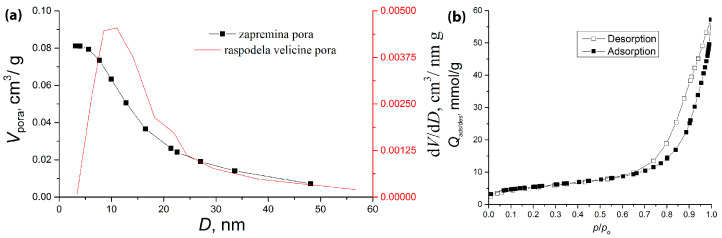
(**a**) Pore size distribution curve and cumulative pore volume of the samples calculated from the desorption branch of the isotherm by the BJH method; (**b**) adsorption and desorption isotherms of the CuAl-LDH@SiO_2_ composite material.

The results of textural properties determination are given in Table 1 and Figure 6.

The porosity of the synthesized SiO_2_ composite was assessed using nitrogen adsorption/desorption porosimeter. The obtained isotherm (Figure 6a) corresponds to type IV [38], which is characteristic of mesoporous silica materials. The sample shows the presence of both macropores and mesopores, as evidenced by the sharp increase in the adsorption isotherm at p/p0 > 0.9 and the presence of a hysteresis loop. The pore size distribution curve (Figure 6a), calculated using the BJH method from the desorption branch, reveals a unimodal pore size distribution, with a predominant pore size of approximately 13.5 nm. The specific surface area, determined using the Brunauer–Emmett–Teller (BET) method, is 19.1 m^2^/g, which is relatively high compared with pure commercial SiO_2_. These results suggest that the synthesized LDH also contributes to the overall surface area of the material [39].

### 2.3. Sorption Experiments

#### 2.3.1. Adsorption Isotherms

**Figure 7:**
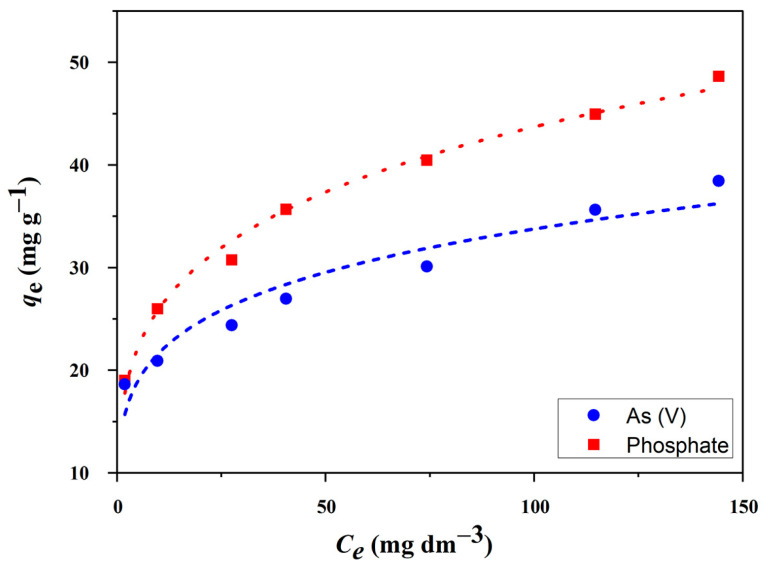
Adsorption isotherms of CuAl-LDH@SiO_2_ for PO_4_^3−^ and As(V).

Batch experiments for the removal of phosphate and As(V) using CuAl-LDH@SiO_2_ as an adsorbent were conducted at pH 4 with a 24 h equilibration period. The results, shown in Figure 7, present the equilibrium adsorption amount (q_e_) as a function of the equilibrium concentration (C_e_) of phosphate and arsenate, with initial concentrations ranging from 2 to 200 mg L^−1^.

The total amounts of adsorbed phosphate and As(V) increased with higher initial concentration. Specifically, the amounts of adsorbed phosphate and As(V) were 48.6 mg PO_4_^3−^ g^−1^ and 38.5 mg As(V) g^−1^, respectively. A strong correlation was observed between the adsorption data and the BET results, suggesting that the increase in the adsorbent’s surface area contributes to the enhanced adsorption capacity. Experimental results further indicated that phosphate and arsenate adsorption on unmodified SiO_2_ had improved adsorption performance compared with its individual components. The inclusion of LDH in the composite provides additional functional properties, thus enhancing its potential for the removal of various pollutants.

The regression coefficients suggest that the Freundlich model is more appropriate for describing the sorption behavior of phosphate and arsenate on CuAl-LDH@SiO_2_, as shown in Table 2. The maximum sorption capacities, calculated using the Langmuir equation, were 44.6 mg PO_4_^3−^ g^−1^ and 32.3 mg As(V) g^−1^, respectively. The high intensity of interactions between the functional groups on the adsorbent surface and the adsorbates is confirmed by the elevated values of the Langmuir constant (*K_L_*), reflecting a higher adsorption affinity compared with pollutants. The correlation coefficients (*R*^2^) for the Langmuir model range from 0.798 to 0.611, while those for the Freundlich model are higher, ranging from 0.992 to 0.937. The values of the parameter n, which range from 1 to 10, indicate that the adsorption process for these anionic species is favorable. Additionally, the Freundlich constant (*K_F_*) serves as an approximate indicator of the adsorption capacity, further confirming the high adsorption capacity of CuAl-LDH@SiO_2_.

#### 2.3.2. Adsorption Kinetic Studies

**Figure 8:**
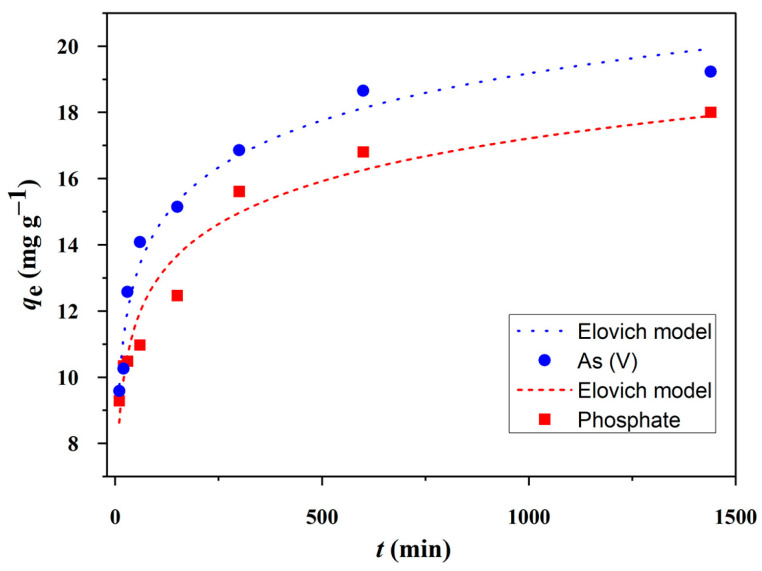
Adsorption of phosphate and arsenate at CuAl-LDH@SiO_2_ as a function of contact time.

The adsorption kinetics of phosphate and As(V) onto CuAl-LDH@SiO_2_ were investigated over a contact time of up to 1440 min. In this experiment, the initial concentrations of phosphate and As(V) were set at 60 mg L^−1^, and the pH of the solutions was adjusted to 4. The removal efficiency of phosphate and As(V) as a function of contact time is illustrated in Figure 8.

The initial phase of phosphate and As(V) adsorption onto CuAl-LDH@SiO_2_ was characterized by rapid uptake. Within the first 10 min, phosphate adsorption reached approximately 52% of its equilibrium capacity, while As(V) adsorption was slightly lower at 50% (Figure 8). This rapid phase was followed by an adsorption phase, which is attributed to intraparticle diffusion and adsorption onto the internal surface sites of the porous adsorbent. Based on the regression coefficient (*R*^2^) values and the standard errors of the model parameters (Table 3), the adsorption kinetics are best described by the linear form of the Elovich model. Originally developed to describe the chemisorption of gases on solid surfaces, the Elovich equation has been widely applied to adsorption systems. The high *R*^2^ values and low chi-square (χ^2^) values confirm that the Elovich model provides the best fit for the experimental data, in agreement with the findings by Farouq et al. [40]. Similar kinetic behavior has been reported for various adsorption systems involving metal ions (Pb^2+^, Cr^3+^, Cr^6+^, Cd^2+^, Cu^2+^, Se^4+^, As^5+^, K^+^, etc.) and dyes, as demonstrated in the study by Wu et al. [41].

**Figure 9:**
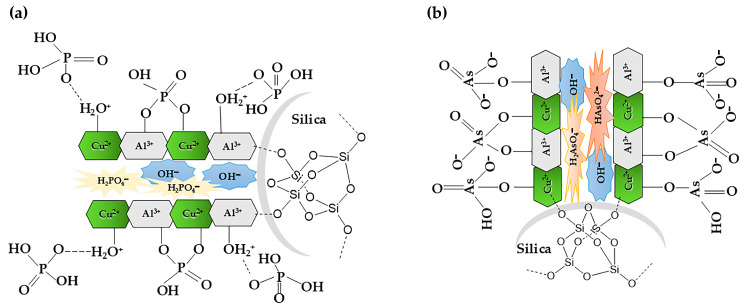
Proposed adsorption mechanism: (**a**) phosphate adsorption; (**b**) arsenate adsorption.

Figure 9 shows the proposed mechanism of phosphate/arsenate adsorption onto the CuAl-LDH@SiO_2_ composite. As depicted, the initial stage of adsorption is rapid, primarily due to the immediate binding of oxyanionic species to the most accessible adsorption sites located on the outer surface and within the larger pores of the adsorbent. The adsorption of these anions is facilitated by multiple mechanisms, including electrostatic attraction, ligand exchange, and ion exchange. The presence of surface M–OH functional groups play a crucial role in enhancing the adsorptive interactions. At pH values below the point of zero charge (pHpzc), the surface hydroxyl groups become protonated, forming –OH_2_^+^ groups. These protonated groups are more readily exchangeable than their neutral –OH counterparts, thereby favoring the adsorption of negatively charged species such as phosphate and arsenate. The observed substitution of surface hydroxyl groups (M–OH) with phosphate and arsenate ions provides strong evidence for a ligand exchange mechanism during the adsorption process [15].

#### 2.3.3. Real Water Matrix Studies

A key concern when using synthetic aqueous matrices is their limited applicability to real-world water environments. Therefore, evaluating new materials using real matrices—such as domestic wastewater effluent, human urine, or swine manure effluent—is essential. Characterizing these matrices (e.g., pH, total dissolved solids, turbidity, competing anions, and dissolved organic matter) is critical for benchmarking phosphorus removal performance across studies. The main challenge lies in balancing the advantages of synthetic matrices—such as known composition and controlled conditions—with the complexity and variability of real samples, which are more difficult to fully characterize [42].

**Figure 10:**
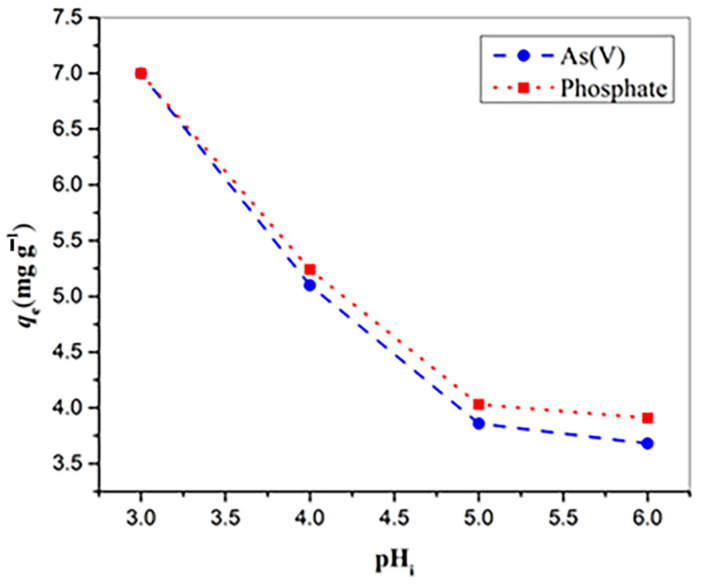
Adsorption of phosphate and arsenate at CuAl-LDH@SiO_2_ on the real water matrix sample, and effect of initial pH. As(V)/phosphate concentration of 7 mg L^−1^.

The results obtained from experiments using real water matrices are shown in Figure 10. The water used as a matrix was sampled from the Danube River near the Vinča settlement in Belgrade. Water samples with a concentration of 7 mg L^−1^ were prepared by spiking arsenate/phosphate into the real water sample. Arsenate/Phosphate removal was performed on four water samples with adjusted pH values of 3, 4, and 5, and 6.20 mg of adsorbent was added to each 20 mL of sample, and after 24 h on a shaker, the samples were analyzed for arsenate/phosphate content.

The four selected pH values for the real water sample were set below the pHpzc value (6.51). As shown in Figure 10, the lowest measured adsorption of arsenate and phosphate occurred at pH 6, with values of 3.68 and 3.91 mg g^−1^, respectively. At pH 5, there was a slight increase in adsorption, reaching 3.86 mg g^−1^ for arsenates and 4.03 mg g^−1^ for phosphates. At pH 4, arsenate uptake increased to 5.1 mg g^−1^ and phosphate uptake to 5.24 mg g^−1^. Finally, at pH 3, the entire amount of arsenate/phosphate was removed from the water. Experiments using the real water matrix demonstrated the material’s strong ability to effectively remove arsenate and phosphate from real water samples, indicating that it could be applicable outside of laboratory conditions.

Layered double hydroxides (LDHs) are gaining attention for water pollutant removal, but their application remains largely limited to mono-component, lab-scale studies. To move toward commercial use, research must focus on sustainable, low-cost strategies, multi-component systems, and real wastewater conditions. Advanced optimization and desorption methods are needed to improve performance, enable adsorbent reuse, and recover pollutants. A deeper understanding of removal mechanisms and testing in both batch and continuous systems with real effluents is essential for practical implementation [43].

#### 2.3.4. Comparative Discussion of Adsorption Results Using LDH-Based Adsorbents

Table 4 presents the adsorption capacities achieved for arsenate and phosphate using layered double hydroxides (LDHs) with various metal ion combinations, as reported in previous studies. The adsorption capacities of CuAl-LDH are comparable with, or even higher than, those reported in the literature (Table 4).

While many studies focus on removing single pollutants from aqueous solutions, real wastewater matrices contain various compounds that can either enhance or inhibit the sorption of the target pollutant [54]. Research on pollutant removal from environmental wastewater is limited. One study showed that ZnAl-Cl-LDH removed 90% of color and reduced COD from wastewater in an industrial dyeing plant [55]. However, it provides little data on wastewater characteristics or how other chemicals impact LDH behavior. Further studies are needed to explore LDH performance in real environmental wastewater, beyond synthetic models, as noted in other reviews on LDH-based pollutant removal [56]. In paper [53], LDH-Cl was utilized to treat a natural groundwater sample containing 231 mg/L arsenic. The findings indicated that LDH-Cl possessed a superior adsorption capacity for As(V). The adsorption experiments were carried out using the untreated and undiluted water sample, maintaining its original pH.

LDHs can be supported on biochar to form composite adsorbents. In this study, however, the CuAl-LDH was deposited on silica, resulting in significantly higher removal efficiencies compared with those reported in the literature. Also, to the best of the authors’ knowledge, not many studies include the combination of Cu Al LDH for the treatment of wastewater generated during the application of the technology. Lowering the concentrations of these harmful ions to levels that are considered safe for discharge into industrial wastewater streams is critical, ensuring they do not cause significant environmental or health impacts.

As part of the circular (bio)economy and sustainability framework, the development of novel and advanced LDH composites incorporating biochar or biomass-derived carbon, along with the identification of the optimal ratio between the components, has become a dynamic area of research.

## 3. Materials and Methods

In this study, CuAl-LDH particles were synthesized using the co-precipitation method in the presence of surface-modified silica particles, resulting in a composite material with enhanced adsorption properties.

### 3.1. Materials

The starting chemicals, copper nitrate (Cu(NO_3_)_2_·3H_2_O), GLYMO, polyethyleneimine, aluminum nitrate (Al(NO_3_)_2_·9H_2_O), and sodium hydroxide (NaOH), were obtained from Sigma-Aldrich Company, Germany; silica particles (Poraver, Germany) were used without further purification. Deionized water was used as a solvent throughout this study.

### 3.2. Modification of Silica with GLYMO Silane (SiO_2_/GLYMO)

The surface of the adsorbent is one of the most important factors in the adsorption process as it is directly related to the adsorption capacity and the efficiency of contaminant removal. In order to synthesize an efficient sorbent with enhanced surface functionality and an increased number of active sites for the removal of impurities from water, the surface of the silica particles was first modified with GLYMO silane (3-glycidyloxypropyltrimethoxysilane), followed by polyethyleneimine (PEI). The process of SiO_2_/GLYMO formation was carried out as follows: In the first step of the modification, 30 g of silica particles were placed in 250 mL of toluene in a three-necked flask. Then, 1.5 g of GLYMO silane was added to the mixture, placed on a magnetic stirrer, and heated to 70 °C for 12 h. The resulting material was washed three times with 20 mL of toluene. In the second part of the modification, material from the first step was placed into another vessel with three necks and on a magnetic stirrer. Afterwards, 5 g of PEI was added, placed on a stirrer, and heated to 80 °C for 8 h. In order to remove the particles from the beaker more easily, 2 mL of dimethylformamide was added at the end of the reaction.

### 3.3. Synthesis of CuAl-LDH and Preparation of CuAl-LDH@SiO_2_ Composites

The co-precipitation method was used to synthesize CuAl-LDH. First, aqueous solutions of Cu(NO_3_)_2_·3H_2_O and Al(NO_3_)_3_·9H_2_O, in a molar ratio of Cu^2+^/Al^3+^ = 2:1, were prepared and mixed in 30 cm^3^ of deionized water. The resulting solution was added to the reactor equipped with a magnetic stirrer in the presence of modified silica (SiO_2_/GLYMO). Based on preliminary results of synthesized composites at different LDH/SiO_2_ ratios, increasing the ratio above 2:1 did not achieve the effect of increasing the specific surface area. A composite material that had lower material costs and was more acceptable from an economic point of view was selected. Therefore, CuAl-LDH@SiO_2_ composites were prepared at mass ratios of LDH/SiO_2_ = 2:1. The metal solutions and silica particles were homogenized for about 10 min. Then, the co-precipitation reaction was achieved at pH 10 by the dropwise addition of 1 M NaOH. The precipitates were aged for 24 h and separated by centrifugation (6000 rpm/10 min), washed with deionized water, and finally dried at 80 °C for 24 h. A light blue color powder was obtained.

### 3.4. Material Characterization

Characterization of CuAl-LDH@SiO_2_ was determined by MIRA3 TESCAN (Oxford, UK) field-emission scanning electron microscopy (FESEM). The software Image-Pro Plus 4.0 (Media Cybernetics, Rockville, MD, USA) was used, and its tools were employed for morphological characterization. The JEOL 5800 JSM (Oxford, UK) used electronic dispersive spectroscopy (EDS) to perform elemental analysis on interfaces. The physicochemical characterization of materials was investigated by an X-ray diffraction analysis (XRD). The specific surface area of samples was calculated using the Brunauer–Emmett–Teller (BET) method.

### 3.5. Adsorption Experiments

At a preset solution pH, the adsorption capacity of the produced composites for binding phosphate and arsenate was examined under a variety of conditions, such as solutions of different concentrations and adsorption periods. A series of previously prepared pollutant solutions was filtered to ascertain the kinetic parameters. Equilibrating 2–200 mg L^−1^ solutions with 1 g L^−1^ of adsorbent at a pH of 4 for 24 h at room temperature was the initial step in the adsorption process. A digitally adjustable Windaus Labor Technik shaker at 200 rpm was used for shaking in the batch system studies. Following equilibration, a 0.22 µm PTFE syringe filter was used to filter the eluent. A UV–Vis spectrophotometer 1800, Shimadzu, Kyoto City, Japan, was used to examine the phosphate concentration in the collected filtrate. As(V) concentration was determined using an atomic absorption spectrometer (Perkin-Elmer AAnalyst 700, Waltham, MA, USA) using a flame technique (FAAS). We used 0.1 M KOH and 0.1 M HNO_3_ solutions to change the solution’s original pH. The widely used Langmuir and Freundlich isotherms were used to assess the adsorption equilibrium.

The experimental data were fitted to the Langmuir (Equation (1)) and Freundlich (Equation (2)) isotherm models [57]:

(1) $$q_{e}=\frac{\left(Q_{m}K_{L}C_{e}\right)}{\left(1+K_{L}C_{e}\right)}$$

(2) qe=KFC1n

where *q_e_* is the equilibrium adsorption capacity of pollutant on an adsorbent (mg g^−1^), *C_e_* is the equilibrium pollutant concentration in a solution (mg L^−1^), *Q_m_* is the maximum capacity of the adsorbent (mg g^−1^), *K_L_* is the Langmuir adsorption constant (L mg^−1^), *K_F_* is the Freundlich adsorption constant (mg^(1−1/n)^ L^1/n^ g^−1^), and *n* is the Freundlich constant correlated with the adsorption capacity and adsorption intensity, respectively.

The kinetic data were analyzed using pseudo-first-order (PFO, Equation (3)), pseudo-second-order (PSO, Equation (4)), intra-particle diffusion (Equation (5)), and Elovich (Equation (6)) kinetic models [57,58]:

(3) $$q_{t}=q_{e}\left(1-e^{-k_{1}t}\right)$$

(4) $$q_{t}=\frac{k_{2}{q_{e}}^{2}t}{1+k_{2}q_{e}t}$$

(5) $$q_{t}=k_{\mathrm{int}}t^{0.5}+C$$

(6) $$q_{t}=\frac{1}{\beta }\ln(a\beta )+\frac{1}{\beta }\ln t$$

where *k*_1_ (min^−1^) is the pseudo-first-order rate constant, *k*_2_ (g mg^−1^ min^−1^) is the pseudo-second-order rate constant of adsorption, *k_int_* is the intraparticle diffusion rate constant (mg g^−1^ min^−0.5^), *C* is the intercept, *α* (mg g^−1^ min^−1^) is the initial adsorption rate constant, *β* (g mg^−1^) is the parameter related to the extent of surface coverage and activation energy of the Elovich equation, *q*_e_ (mg g^−1^) is the equilibrium adsorption capacity, and *q*_t_ (mg g^−1^) is the adsorption at time *t* (min).

### 3.6. pH_PZC_ Determination and Texture Properties

The point of zero charges (pH_PZC_) refers to the pH at which the total positive and negative charges on the particle surface are equal, meaning the net surface charge is zero. To determine the pH_PZC_ value, an HI-2210-02 bench-top pH meter from (Szeged, Hungary) with an accuracy of ±0.01 was used. Solutions with pH values ranging from 2 to 12 were prepared using KNO_3_ at concentrations of 0.01 and 0.001 M. An amount of 20 mL of each KNO_3_ solution was allowed to equilibrate with 20 mg of adsorbent for 24 h. The initial pH of each sample was adjusted, and after 24 h, the final pH was measured and recorded [57].

Nitrogen adsorption–desorption isotherms were determined using a Micromeritics ASAP 2020 instrument. Samples were degassed at 100 °C for 9 h under reduced pressure. The total pore volume (V_total_) was given at *p*/*p*_0_ = 0.998. The volume of the mesopores (V_meso_) was calculated according to the Barrett–Joyner–Halenda (BJH) method from the desorption branch of isotherm. The average pore diameter (D_sr_) was calculated according to the Barrett–Joyner–Halenda (BJH) method from the desorption average pore width (4V/S).

Isotherm modeling showed that the Freundlich model better described the sorption behavior, with Freundlich constants (KF) of 15.4 L mg^−1^ for PO_4_^3−^ and 13.9 L mg^−1^ for As(V) and n values of 4.41 and 5.18, respectively—both within the favorable adsorption range of 1 to 10.

## 4. Conclusions

In this study, a novel composite material comprising Cu-Al layered double hydroxides (CuAl-LDH) supported on modified silica particles was successfully synthesized and thoroughly characterized for the efficient removal of phosphate and arsenate ions from aqueous solutions. The synthesis strategy was designed in alignment with the Sustainable Development Goals, offering an environmentally responsible and effective approach to wastewater treatment. The surface of silica was modified using 3-glycidyloxypropyltrimethoxysilane (GLYMO) and polyethyleneimine (PEI) creating an ideal platform for the deposition of CuAl-LDH. Characterization through XRD, SEM, FTIR, and BET confirmed successful modification and composite formation, revealing a porous structure with enhanced surface area and active sites favorable for adsorption. Adsorption experiments demonstrated high removal efficiencies, with maximum adsorption capacities reaching 44.6 mg g^−1^ for phosphate and 32.3 mg g^−1^ for arsenate at pH 4. The point of zero charge (pH_pzc_) of 6.51 further validated the adsorbent’s suitability for anionic species. The equilibrium adsorption capacities, calculated using the Langmuir isotherm model, were found to be 44.6 mg·g^−1^ for phosphate (PO_4_^3−^) and 32.3 mg·g^−1^ for arsenate (As(V)) at 25 °C. Isotherm modeling showed that the Freundlich model better described the sorption behavior, with Freundlich constants (K_F_) of 15.4 L mg^−1^ for PO_4_^3−^ and 13.9 L mg^−1^ for As(V) and n values of 4.41 and 5.18, respectively—both within the favorable adsorption range of 1 to 10. Beyond its excellent adsorption performance, this composite material supports circular economy principles by incorporating waste-derived components and reducing environmental impact. This research underscores the significance of integrating bio-based materials, minimizing energy input, and promoting sustainable innovation in wastewater purification technologies. Overall, the CuAl-LDH@SiO_2_ composite represents a promising advancement in water treatment solutions, offering practical implications for pollutant remediation and resource recovery while contributing to global environmental protection efforts.

## Figures and Tables

**Table 1:** Textural properties of CuAl-LDH@SiO_2_.

Sample	*S_p_*, m^2^/g	*V_total_*, cm^3^/g	*V_meso_*, cm^3^/g	*D_sr_*, nm	*D_max_*, nm
SiO_2_	3.5–9.5	–	–	–	–
CuAl-LDH@SiO_2_	19.1	0.0823	0.0812	13.5	11.0

*S_p_*—Specific surface area; *V_total_*—total pore volume; *V_meso_*—mesopore volume, pore ranged between 2.0 and 300 nm, *D_sr_*—average pore diameter; *D_max_*—diameter of the most abundant pores.

**Table 2:** Parameter values of different types of adsorption isotherm models fitted to the experimental results for PO_4_^3−^ and As(V) adsorption on the CuAl-LDH@SiO_2_ sample (pH—4; adsorbent—1 g L^−1^; reaction time—24 h).

Model	Parameter	Pollutants	
		PO_4_^3−^	As(V)
Langmuir	*Q_m_* (mg g^−1^)	44.6	32.3
	* K_L_* (L mg^−1^)	0.183	0.396
	*χ* ^2^	31.5	29.4
	*R* ^2^	0.798	0.611
Freundlich	*K_F_* (L mg^−1^)	15.4	13.9
	*n*	4.41	5.18
	*χ* ^2^	1.33	4.74
	*R* ^2^	0.992	0.937

**Table 3:** Parameter values of the kinetic models fitted to the experimental results for PO_4_^3−^ and arsenate adsorption on the CuAl-LDH@SiO_2_ sample (pH—4; adsorbent—1 g L^−1^; *Ci* = 60 mg L^−1^; reaction time—24 h).

Model	Parameter	Pollutants	
		PO_4_^3−^	As(V)
Pseudo-first-order	*q_e_* (mg g^−1^)	14.8	17.0
	*k*_1_ (min^−1^)	0.0591	0.0521
	*χ* ^2^	7.06	4.05
	*R* ^2^	0.524	0.768
Pseudo-second-order	*q_e_* (mg g^−1^)	16.0	18.06
	*k*_2_ (g mg^−1^ min^−1^)	0.00512	0.00433
	*χ* ^2^	3.66	1.52
	*R* ^2^	0.753	0.913
Intraparticle diffusion	*k_int_* (mg g^−1^ min^−0.5^)	0.264	0.271
	*C*	9.27	10.7
	*χ* ^2^	1.13	2.77
	*R* ^2^	0.924	0.841
Elovich	*α* (mg g^−1^ min^−1^)	19.0	24.0
	*β* (g mg^−1^)	0.536	0.489
	*χ* ^2^	0.637	0.404
	*R* ^2^	0.957	0.978

**Table 4:** A comparative analysis of the adsorption capacities of LDH-based adsorbents.

Adsorbent	Pollutant	*C*_i_, mg dm^−3^	*q*_m_, mg g^−1^	*k*, g mg^−1^ min^−1^	Reference
Mg/Mn/Fe-LDH	As(V)	4–270	32.2	0.019	[9]
Fe/Mn-C-LDH	As(V)	100	22.2	0.0077	[44]
Mg/Fe-modified biochar	As(V)	1	3.6	0.02	[45]
PS-La-LDH	Phosphate	5–50	34.2	0.001	[46]
Cu-Zn-Fe LDH	Atrazine	5–30	21.85	-	[28]
Cu-TiO_2_	As(V)	3	19.72	0.056	[47]
NCMP@MgFe-Zr	Phosphate	25	9.6	0.017	[48]
Mg-Al-CO_3_ LDH	Phosphate	10–120	54.9	0.006	[49]
FeNP/MFC	As(V)	0.14–10.68	2.46	0.0375	[50]
Zn-Al LDH	Phosphate	5–200	35.9	0.055	[51]
MgAl-NO_3_ LDH	Phosphate	100	64.1	0.005	[52]
LDH-CO_3_ LDH-Cl	As(V)	0.2	44.7 88.3	- -	[53]
FeAl-LDH@SiO_2_	As(V) Phosphate	100 100	46.0 58.9	- -	[15]
CuAl-LDH@SiO_2_	As(V) Phosphate	2–200 2–200	32.3 44.6	0.00433 0.00512	This work

## Data Availability

The original contributions presented in this study are included in the article. Further inquiries can be directed to the corresponding author.

## Abbreviations

The following abbreviations are used in this manuscript:

LDHs	Layered double hydroxides
PEI	Polyethyleneimine
XRD	X-ray diffraction
SEM	Scanning electron microscopy
EDS	Electronic dispersive spectroscopy
BET	Brunauer–Emmett–Teller
GLYMO	3-glycidyloxypropyltrimethoxysilane
FESEM	Field-emission scanning electron microscopy
FAAS	Flame atomic absorption spectrometry
PFO	Pseudo-first-order
PSO	Pseudo-second-order
